# Social Virtual Reality (VR) Involvement Affects Depression When Social Connectedness and Self-Esteem Are Low: A Moderated Mediation on Well-Being

**DOI:** 10.3389/fpsyg.2021.753019

**Published:** 2021-11-30

**Authors:** Hyun-Woo Lee, Sanghoon Kim, Jun-Phil Uhm

**Affiliations:** Sport Experience Management Lab, The Department of Health and Kinesiology, Texas A&M University, College Station, TX, United States

**Keywords:** social VR game, involvement, social connectedness, well-being, depression, self-esteem

## Abstract

While social interaction and play in a VR environment are becoming ever more popular, little is known about how social VR games affect users. The purpose of this study was to elucidate the role of several contingent factors in social VR games by modeling the relationships between involvement, well-being, depression, self-esteem, and social connectedness. A conditional process-moderated mediation model of the measured variables was analyzed with 220 pieces of collected data. The result showed that: (1) the direct effect of involvement on well-being was significant, and (2) the index of moderated mediation involving depression, self-esteem, and social connectedness was significant. We conclude that high levels of involvement in social VR games by socially isolated users with low self-esteem can negatively affect their well-being. The findings of this study contribute in several ways to our understanding of the effect of social VR games upon users and provide important practical implications.

## Introduction

Social connection through new and improved technology-mediated communication has become a mainstay in an ever-changing society, and innovative extended reality technologies, such as augmented reality (AR) and virtual reality (VR), have the potential to advance social connection from face-to-face and 2D interfaces into a lively and interactive 3D virtual environment. Moving beyond existing applications, the development of new multi-user interfaces is expected to accelerate social connection *via* virtual environments. Even so, the social aspects of multi-user VR games have been overlooked despite the rapidly growing marketing of such products (Uhm et al., 2020).

There is a consensus among social scientists that psychological support from social relationships enhances well-being (Lee et al., 2008; Jose et al., 2012; Pressman et al., 2019). Conversely, lack of social connection is a risk factor for social isolation, depression, and premature mortality (Cacioppo et al., 2010; Matthews et al., 2016). When it comes to new media environments, social relationships are not without their risks. For instance, existing evidence indicates negative use of technology-mediated communication can lead to antisocial behaviors, such as aggression, loneliness, gaming addiction, cyberbullying, and more (Valkenburg et al., 2016; Holt-Lunstad, 2018).

Unlike regular massive multiplayer online games for computers and consoles, a social VR game allows players to interact in a virtual world using a combination of a head-mounted display, audio, and input devices to achieve a higher level of immersion. More specifically, such games require a player to wear a head-mounted device and controllers that track their physical movements and enable them to manipulate their avatar’s motions using their body while viewing and interacting with a fully virtual landscape. Additionally, social VR games give users the experience of meeting other players and communicating with them in virtual environments. The game Rec Room, for example, allows players to meet one another and physically engage in various games (e.g., dodgeball and disc golf) together.

Nevertheless, there remains a paucity of evidence on the interactions between users’ social VR involvement and their psychological aspects. Thus, the purpose of this study was to elucidate the role of several contingent factors in social VR games by modeling the relationships between involvement, well-being, depression, self-esteem, and social connectedness in social VR. Specifically, we used a working definition of social VR as VR content with multi-user functions where users can simultaneously interact with each other in the VR environment. As we applied the literature of involvement to operationalize social VR use, depression was identified as an affective mediator of the influence of involvement on well-being. To capture conditional effects of individual sense of self-worth and closeness/connectedness with others, self-esteem and social connectedness in social VR were measured as moderators.

## Literature Review and Hypothesis Development

### Involvement and Well-Being

Involvement pertains to an individual’s perceived personal importance and the significance of an attitude object to their innate interests, values, and needs (Zaichkowsky, 1985). Historically, the concept of involvement has attracted a lot of interest across various disciplines, and it has been also used to explain user experiences in virtual settings. For example, Cowan and Ketron (2019) explored how much involvement impacts users’ imagination and perceived interactivity in a virtual environment. Jin (2009) has also investigated the degree to which consumer involvement in products impacts their attitude toward those products and pleasure in a virtual environment, which has provided useful insights into virtual retailing. Taking these prior concepts of involvement as a basis, the current study defines involvement as the gamer’s level of interest in a social VR game.

Involvement has the potential to be a critical emerging concept in the understanding of VR gaming and its effects on well-being. According to Diener et al. (2002), well-being can be described as experiencing happiness (which includes life satisfaction and positive attitude) that can be attained by achieving a certain state – for example, meeting a need or a goal. A person with a sense of well-being is “blessed with a positive temperament, tends to look on the bright side of things, and does not ruminate excessively about bad events, has social confidants, and possesses adequate resources for making progress toward valued goals” (Diener et al., 1999, p. 295). The body of online gaming literature investigating the relationships between game involvement and well-being is currently weakened by inconsistent findings.

In reviewing previous works, some recent research indicated that social game use can negatively impact several well-being constructs. For instance, Williams and Merten (2008) stated that both male and female online social gamers report significantly higher prevalence of depression and substance addiction than is typical within the United States population. Some studies have also suggested a connection between engaging in online games and symptoms of depression, anxiety, poor self-esteem, and issues in interpersonal relationships (Morgan and Cotten, 2003; Lo et al., 2005; Stetina et al., 2011). Likewise, online game players have been noted to exhibit elevated physical problems, such as health and sleep issues, personal life problems, and academic or professional issues (Smyth, 2007).

Even though researchers have asserted relationships between social gaming and a wide range of negative outcomes, effect sizes are small, much of the research is correlational, and the majority of respondents report few or no negative effects (Smyth, 2007; Liu and Peng, 2009). In this regard, another line of research has reported that engagement in both social games and VR games is associated with some significant benefits. For instance, Mandryk et al. (2020) contended that involvement in social games can cultivate well-being by providing players with an outlet for daily stressors, thus allowing them to cope with life’s obstacles, practice emotional regulation, and pursue meaningful social interaction.

In terms of VR gaming, Singh et al. (2017) stated that interactive VR games can enhance psychological well-being for users, who may benefit from using this technology because it promotes adherence, motivation, and involvement in physical activity. Furthermore, the effects of gaming on well-being are an important factor in the motivations behind game play, such as participating for pleasure rather than playing for achievement or due to obsession (Lafreniere et al., 2009; Carras et al., 2017). Considering the beneficial social interaction and physical activity-related impacts of social VR games, involvement in social VR games should result in high levels of well-being. On this basis, we developed the following hypothesis:

*Hypothesis* 1: Involvement in social VR game positively affects well-being.

### The Mediating Role of Depression

Over the past several years, research has reported that engaging in excessive video game playing leads to psychological and social challenges that make it hard to cope with day-to-day life. Studies have supported the notion that although playing social games can boost mental health and reduce stress (e.g., LaRose et al., 2003), excessive play can have negative emotional consequences (e.g., King and Delfabbro, 2016; Wang et al., 2019; Lavoie et al., 2021). Among the reported negative emotional consequences, previous studies found that there was a relationship between depression and online gaming. For instance, Loton et al. (2016) contended that video game addicts exhibited poorer mental health and cognitive functioning, along with greater emotional challenges, such as increased depression and anxiety. Similarly, prior research has reported, for example, that highly involved gamers were at a higher risk of depression (Wang et al., 2019). Moreover, Maras et al. (2015) found that the amount of time spent on games was correlated with higher levels of depression. As such, many studies have shown that excessive involvement in games is strongly associated with depression.

Traditionally, it has been argued that well-being is inversely related to depression (Howard et al., 1993; Callahan et al., 2006). Studies found that those with fewer depressive disorders enjoy a greater degree of well-being (Chaplin, 2006), and others provided evidence that a decrease in symptoms of maladjustment creates fertile ground for well-being to thrive (Howard et al., 1993; Callahan et al., 2006). Likewise, with gaming, because depression is reported to have negative associations with mental health (Cardak, 2013), it is expected that social VR gamers’ level of depression will have a negative relationship with their well-being. Based on these considerations, we posited that depression mediates the relationship between involvement in social VR games and well-being. In addition, while we set the mediating role of depression between involvement in social VR games and well-being, we also identified that the mediation effect can be conditional based on personal characteristics, such as self-esteem and social connection.

### The Moderating Role of Self-Esteem and Social Connection

As discussed in previous studies, an individual’s involvement in games induces positive psychological responses (i.e., well-being) in some studies and negative psychological responses (i.e., depression) in others, confirming inconsistent results regarding the effect of such activities. Such conflicting outcomes cause confusion among scholars and practitioners. This may, however, arise from the studies not reflecting idiosyncratic differences inherent to the individuals who are playing the games or because the platform of the game itself is not taken into consideration. In general, mental health is found to be significantly affected by an individual’s lifestyle or psychological elements, and mental health as it relates to gaming is no exception. For instance, Loton et al. (2016) claimed that the effect of time spent in gameplay on stress reduction differed significantly depending on the individual’s coping level.

Based on existing research findings, different results present themselves depending on individual differences (e.g., lifestyle) in the relationship between social VR game involvement and mental health factors, such as depression and well-being. In previous studies, people with higher self-esteem – representing psychological resources – had lower levels of depression and that the two factors have a negative correlation (Lin, 2015). Williams and Galliher (2006) found that individuals with high levels of social connection in daily life had low levels of depression and high self-esteem. As such, the influence on an individual’s mental health may vary according to individual characteristics or lifestyle. Applying, it is reasonable to assume that the influence of social VR game involvement on depression can present differently, depending on the social connection provided by social VR games.

**Figure 1:**
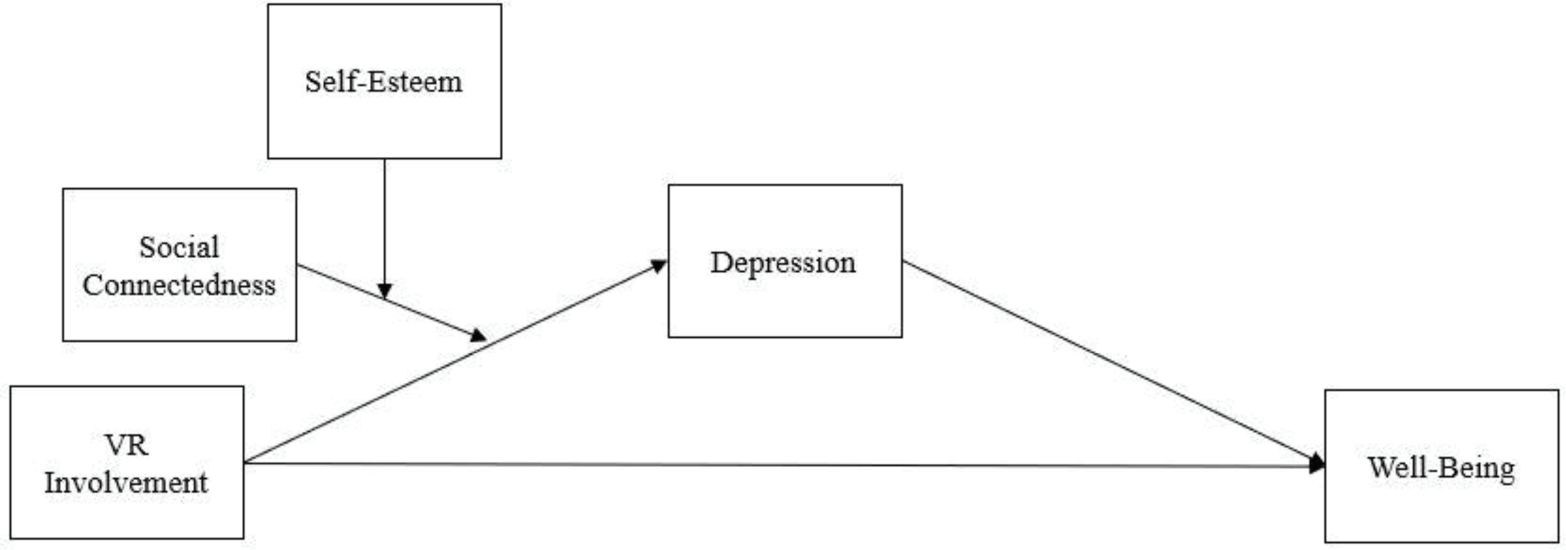
Research model.

In the current study, we considered self-esteem to be a moderator between involvement in social VR games and depression, based on the assumption that when involvement in social VR games evokes a feeling of depression, the perceived depression decreases if self-esteem is high. In this regard, the depression, low self-esteem, and low levels of well-being can be seen as being closely related to each other. However, each represents a distinctly different individual state, and previous studies have verified that the constructs were distinctive in a statistical model assessment (e.g., Lin, 2015). We also suggest social connection as an additional moderator, inferring that the relationship between involvement in social VR, depression, and well-being differ by the level of self-esteem and social connection. Combining these existing studies and assumptions, we propose a moderated mediation (Hayes, 2018) by establishing the following hypothesis. Figure 1 presents the combined effects, posited in the hypotheses, as a path model.

*Hypothesis* 2: In the relationship in which depression mediates involvement and well-being in social VR games, self-esteem and social connection will moderate the relationship between involvement and depression.

## Materials and Methods

### Participants and Procedures

Participants for this study were users of social VR games in the United States. We collected and recorded data using an online survey tool. Of the 300 contacts, a total of 220 responses (response rate: 73.33%) were obtained: 139 males (63.19%) and 81 females (36.81%). Participants’ average gameplay per week was 9.10h, and their average age was 37.56. The academic completion levels of participants included six (2.73%) high school graduates, nine (4.09%) with some college but no degree, 13 (5.91%) with an associate’s degree (2years), 130 (59.09%) with a bachelor’s degree (4years), 59 (26.82%) with a master’s degree, and three (1.36%) with a doctoral degree. In terms of total household income, 10 (4.55%) reported less than $10,000, 27 (12.27%) reported $10,000-29,999, 51 (23.18%) reported $30,000–49,999, 51 (23.18%) reported $50,000–69,999, and 81 (36.82%) reported household income of $70,000 or more. Among those who indicated that they typically enjoyed sport-related content, the majority of the users mentioned VR action games (46%), followed by adventure (16%), sports (15%), casual (14%), and strategy (9%). Approval for this research was granted by the Texas A&M University Institutional Review prior to the collection of data.

### Measures

Among several other scales to measure the variable we focused on, we used scales that included items that aligned with the needs of our research. We also adopted the scales for which the reliability and validity have been adequately assessed in previous studies. The selected items were revised and reworded for the social VR game context based on experts’ suggestions according to content relevance, representativeness, and item clarity. Specifically, we used 10 items from the Revised Personal Involvement Inventory (RPII), formulated by Zaichkowsky (1994), to measure involvement in a social VR game. Examples include “To me, social VR games are important” and “To me, social VR games are exciting.” Eight items from the new well-being measures scale developed by Diener et al. (2010) measured the level of well-being of the social VR game users. Examples of new well-being scales are “I am engaged and interested in my daily activities,” “I actively contribute to the happiness and well-being of others,” and “I am optimistic about my future.” To measure depression, we adopted 20 items from the Center for Epidemiologic Studies depression scale (CES-D; Radloff, 1991). Examples of CES-D items were as follows: “I felt depressed,” “I felt people disliked me,” and “I felt lonely.” Self-esteem perceived by social VR game users was measured using 10 items from the global self-esteem scale (Rosenberg, 2015). Examples of global self-esteem include “On the whole, I am satisfied with myself” and “I feel that I have a number of good qualities.” Additionally, 20 items from the Social Connectedness Scale-Revised (SCS-R; Lee et al., 2001) were used to measure the degree to which social VR game users perceived their connectedness with other users in daily life. Examples include “I am able to connect with other people,” “I fit in well in new situations,” and “I feel comfortable in the presence of strangers.” All aspects were measured using a 7-point Likert-type scale. Cronbach’s alpha for the sample was satisfactory: 0.86 for involvement, 0.84 for well-being, 0.92 for depression, 0.73 for social connection, and 0.80 for self-esteem.

### Statistics and Data Analysis

Before testing the hypotheses, we checked for correlations and ensured that assumptions of normality were met in the data. To test our hypotheses, we used a conditional process model to test the moderated mediation (Model 11 in PROCESS; Hayes, 2018). In this analysis, we set involvement as the independent variable, well-being as the dependent variable, depression as the mediator, and social connectedness in social VR and self-esteem as moderators. Moderated mediation model describes when the moderation of the indirect effect of one variable is dependent on the second moderator (Hayes, 2018). This can occur in models with a three-way interaction between an independent variable and two moderators. The index of moderated mediation is an inference of whether the moderation of the indirect effect of an independent variable on the dependent variable by the first moderator is moderated by the second moderator. Applying moderated mediation to our research model, we hypothesized a three-way interaction between involvement, social connectedness, and self-esteem and examined whether the moderation of the indirect effect of involvement on well-being (through depression) by social connectedness is moderated by self-esteem. Following Hayes’ (2018) criteria, to ensure the probed points are within the range of observed data (cf. standard deviations are subject to this case), we set the percentiles as 16 for low self-esteem and social connectedness levels, 50 as the median, and 84 for high self-esteem and social connectedness levels.

## Results

Descriptive statistics of the variables are reported in Table 1. Measures were deemed normally distributed considering the univariate statistics of the practical data (Cain et al., 2017). Path coefficient and confidence interval estimates of the model are reported in Table 2. Supporting H1, the direct effect of involvement on well-being was significant. All predictors including interaction effects on depression were significant (*p*s<0.05).

**Table 1:** Descriptive statistics and correlations for study variables (*N*=220).

	1	2	3	4	5
1. VR involvement	1				
2. Depression	−0.03	1			
3. Self-esteem	−0.06	−0.74^***^	1		
4. Social connectedness	−0.06	−0.80^***^	0.78^***^	1	
5. Well-being	0.39^***^	−0.15^*^	0.14^*^	0.09	1
*M*	4.02	3.1	2.6	2.8	4.07
*SD*	0.75	1.06	1.08	0.64	0.67
Skewness	−0.77	−0.56	0.8	0.67	−1.23
Kurtosis	0.62	−0.83	−0.41	0.5	2.75

* *p*<0.05;

*** *p*<0.001.

**Table 2:** Path estimates.

Predictors	*B*	*SE*	*p*	LLCI	ULCI
DV: Depression (*R*^2^ =0.73)					
VR involvement	2.93	0.65	<0.001	1.65	4.20
Self-esteem	2.49	0.99	0.01	0.53	4.45
Social connectedness	3.07	0.95	<0.01	1.20	4.94
VR involvement × Self-esteem	−0.79	0.24	<0.01	−1.26	−0.32
VR involvement × Social connectedness	−1.08	0.23	<0.001	−1.53	−0.64
Self-esteem × Social connectedness	−0.94	0.30	<0.01	−1.54	−0.35
VR involvement × Self-esteem × Social connectedness	0.27	0.07	<0.01	0.12	0.41
DV: Well-Being (*R*^2^ =0.17)					
VR involvement	0.35	0.06	<0.001	0.24	0.46
Depression	−0.09	0.04	<0.05	−0.16	−0.01

DV, dependent variable; SE, standard error; LLCI, lower level of confidence interval; ULCI, upper level of confidence interval.

**Figure 2:**
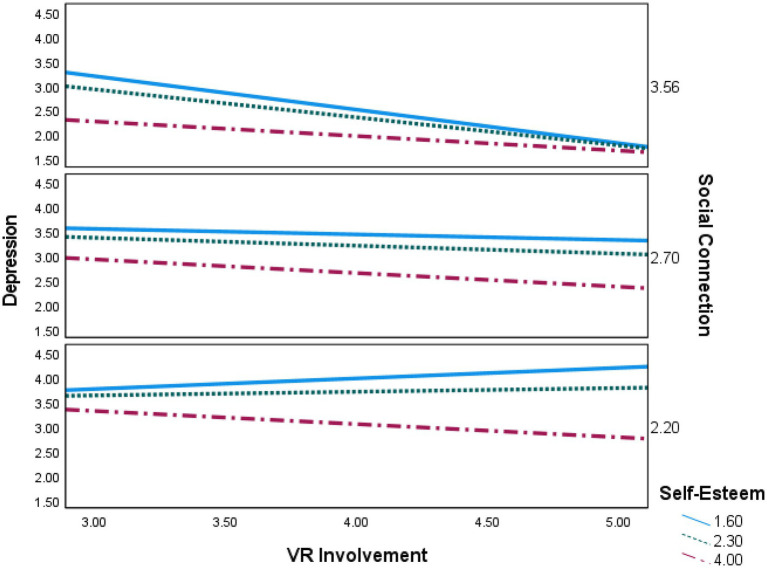
The three-way interaction effect. Solid line represents low self-esteem level at 16th percentile; Dotted line represents medium self-esteem level at 50th percentile; and dash-dotted line represents high self-esteem level at 84th percentile.

The index of moderated mediation was significant (index=−0.023, 95% CIs from −0.046 to −0.004). The mediation effect of depression was partially supported by conditional indirect effects, indicating that the manner in which self-esteem moderated the indirect effect *via* depression is a function of social connectedness in social VR and that the manner in which social connectedness in social VR moderates the indirect effect *via* depression is a function of self-esteem (Hayes, 2018). As reported in Table 3, the indirect effect of social VR involvement *via* depression only had a negative effect when the user had low self-esteem and low social connectedness in social VR. In contrast, for users with moderate to high self-esteem and social connectedness in social VR, the indirect of involvement *via* depression did not negatively affect well-being. Taking a more careful look, from the conditional moderated mediation, the disordinal three-way interaction was most significant for the users with high social connectedness in social VR (index=−0.014, 95% CIs from −0.030 to −0.001). In summary, the results indicate that there was a three-way interaction between involvement, social connectedness, and self-esteem, and the moderation of the indirect effect of involvement on well-being (through depression) by social connectedness is affected by self-esteem. While VR involvement indicated a nonsignificant correlation with depression in general, gamers with low levels of social connection and self-esteem should be cautious in VR involvement as higher VR gaming involvement showed a positive influence on depression in this group. The three-way interaction effect is visualized in Figure 2. We discuss the moderated mediations in more detail in the discussion.

**Table 3:** Conditional indirect effects of social VR involvement on well-being *via* depression.

Self-esteem level	Social connectedness level	Effect	Boot*SE*	BootLLCI	BootULCI
Low	Low	−0.018	0.012	−0.046	−0.002
Low	Median	0.010	0.007	−0.003	0.025
Low	High	0.058	0.026	0.012	0.114
Median	Low	−0.006	0.008	−0.026	0.006
Median	Median	0.014	0.007	0.002	0.030
Median	High	0.049	0.022	0.010	0.094
High	Low	0.023	0.018	−0.010	0.061
High	Median	0.024	0.015	−0.001	0.056
High	High	0.025	0.012	0.005	0.052

Self-Esteem Level: Low=1.6 (16th percentile), Median=2.3 (50th percentile), High=4 (84th percentile); Social Connectedness Level: Low=2.2 (16th percentile), Median=2.7 (50th percentile), High=3.56 (84th percentile). SE, standard error; LLCI, lower level of confidence interval; ULCI, upper level of confidence interval.

## Discussion

In this study, we attempted to examine the impact of social VR involvement on participants’ well-being, going further by identifying depression as a variable that conditionally mediates based upon participants’ level of self-esteem and social connectedness in social VR. The findings of this study confirm and further elucidate the double-edged sword effect of social VR game participation on participants’ well-being by examining moderated mediations between intervening variables. This section offers explanations of the current findings, contributions, and suggestions.

Path estimates revealed that involvement in social VR games can positively affect participants’ well-being, supporting Hypothesis 1, contrary to the findings of many studies in game-related, psychology, and education disciplines (e.g., Groves and Anderson, 2015). Specifically, many scholars reported that excessive involvement in games may lead to game addiction and its harmful psychological and behavioral consequences. Indeed, the World Health Organization recently designated game addiction as a disease, which has resulted in many countries strengthening regulations on games *via* their respective public policies (World Health Organization, 2018). However, our results contradict this conventional negative interpretation, thus possibly providing important practical and academic implications.

Although no direct link between social VR game involvement and users’ well-being has yet been established in sports management, it is encouraging to compare this finding with those reported in other studies using different contexts. For instance, previous studies reported that involvement in physical activity is connected to social outcomes that enhance quality of life, general life satisfaction, and psychological well-being (Penedo and Dahn, 2005). Other studies also demonstrated that involvement in physical activity can meet social needs and lead to benefits such as social support (Mahan et al., 2015). In this regard, as social games enable participants to bolster social connections by interacting with others in a game environment (Beaton and Funk, 2008; Martončik and Lokša, 2016), social VR games can satisfy human needs (i.e., enhance self-esteem) and motivate socially related activity, which in turn improves the overall quality of people’s social lives (i.e., reduce depression; McClelland, 1988). This is imperative for the well-being of VR gamers, which is characterized by high bivariate correlations between self-esteem, social connectedness, and depression (see Table 1).

While our first result showed that involvement in social VR can have a positive effect on participants’ well-being, our second finding demonstrates that participants’ individual characteristics and the social connectedness can change this relationship. The testing of Hypothesis 2 revealed that the mediation effect of depression was partially supported by conditional indirect effects involving social connectedness in social VR and self-esteem. This result indicates how self-esteem moderates the indirect effect of VR involvement on well-being *via* depression is a function of social connectedness in social VR and how the moderation effect of social connectedness is a function of self-esteem. Therefore, we can infer that participation in social VR games may not positively affect participants’ well-being in cases when there are interaction effects of self-esteem and social connectedness.

Although prior research clearly indicates that excessive engagement in computer-mediated games is linked to depression, researchers have devoted relatively less attention to identifying the intervening variables that moderate this relationship. Our results have identified self-esteem as a promising moderator between involvement, depression, and well-being. According to previous studies, people highly engaged in a certain activity (e.g., leisure) are more likely to develop greater self-esteem and self-worth because setting and achieving goals positive impacts their ego (Schmidt and Padilla, 2003). In addition, many studies have indicated that low self-esteem is a primary risk factor for depressive disorders (Orth et al., 2008). With this in mind, our result may be explained by the fact that people with higher involvement in social VR games tend to have lower levels of depression due to greater self-esteem.

Similarly, the results of this study suggest that social connectedness in social VR moderates the relationship between involvement, depression, and well-being. Existing studies indicated that social connectedness can expand momentary repertoires of cognition and behavior, which in turn enhance psychological and social well-being (Fredrickson et al., 2015). Furthermore, based on the phase model of psychotherapy, depressive symptoms must be reduced in order for well-being to be enhanced (Howard et al., 1993). We thus propose that the development of both well-being and depression is subject to sequential relationships, with social connectedness laying the foundation for a reduction in the symptoms of maladjustment.

Many studies have investigated self-esteem and psychological well-being. Theoretical work has underscored the key role of self-esteem in well-being (Kernis, 2003). Empirical studies have demonstrated that self-esteem plays a considerable role in predicting psychological well-being (Sedikides et al., 2007). Other findings have likewise indicated that individuals with higher self-esteem benefit from greater psychological well-being (Baumeister et al., 2003). We conclude from these observations that engagement influences depression through the two moderation effects such that high levels of self-esteem and social connectedness in social VR lead to reducing depression and, in turn, enhance well-being.

Our study provides important academic implications. Our study provides academic implications as we here suggest a model of an individual’s psychological response by analyzing the how personal and social factors influence an individual’s overall well-being when deeply involved in an activity. Prior to this study, research has provided contradictory findings of the effects on a user’s mental health due the participation in social VR games. Thus, it was difficult to make an accurate prediction about how users’ personality traits are associated with Social VR game participation, and what the consequences of that participation may be on their mental health. In this study, the evidence of the psychological mechanisms involved with social VR game participation that we have provided has the potential to provide direction for future research. Our study also provides practical implications useful to social VR game participants. This study suggests that social VR games should be avoided by people who are prone to low levels of social contact and those with low self-esteem. Our findings suggest several courses of action for those who work for the healthcare industry. For instance, as the healthcare industry employs VR and AR for therapy purposes, the results of this study will help to develop effective treatment strategies aimed at different patient groups with specific healthcare needs.

## Limitations and Suggestions

This study has limitations in generalizing its results due to several constraints. First, we did not control the types of social VR games analyzed in this study. There are several types of social VR games, and individual psychology may differ depending on whether a particular type of game requires or induces competition. Therefore, in future research, we suggest conducting a direct experiment to unify the types of games or to conduct a study comparing the effects of cooperative and competitive games. Another limitation was that most of our study subjects were male. Expanding on the above limitations, men and women may enjoy playing different types of social VR games and may respond psychologically to them differently. Therefore, we recommend that follow-up studies feature equal participation between males and females or that a study comparing male and female ratios should be conducted to generalize the results. Our results showed that internal consistency reliability for depression was 0.92, which indicates a potential for item redundancy. As the reason for such a high level may be due to the large number of items measuring depression (Tavakol and Dennick, 2011), more insightful results regarding the measurement of depression could be obtained by employing a scale comprising a smaller number of items or that a context-specific scale could be developed that aligns with the context of social VR games.

Although we have presented interesting findings, including those pertaining to social and personal factors, we believe that a more diverse assortment of psychological factors may be involved. For example, we believe that the consideration of additional variables such as motivation to play a game or the stress level induced by participation in it can provide more implications for social VR games. Therefore, we propose, based on our research model, that subsequent studies consider more diverse variables.

## Conclusion

VR gaming is growing and evolving. It is becoming more accessible through newly developed platforms (e.g., social VR; metaverse), new methods of acquisition (e.g., online app stores), and technological innovations that provide new ways to interact and experience game content with the gaming community (e.g., watching streamers or e-sports tournaments). Social play in a VR environment is becoming ever more popular, with participants playing together with friends, family, colleagues, and strangers. Scholars have suggested that these social interactions benefit players by satisfying essential needs of belonging and connecting with others. However, others have proposed that such engagement is a diluted and dysfunctional social interaction that negatively affects participants by supplanting richer in-person involvement. Recent research consistently illustrates how neither of these interpretations captures the nuances of the reality of social VR gaming and that the benefit or harm derived from social play is predicated on various factors pertaining to the game, the player(s), and the gaming context.

In this paper, we have elucidated the role of several contingent factors in social VR games by modeling the relationships between involvement, well-being, self-esteem, and social connection. Specifically, our results showed that engagement in social VR games by a socially isolated user with low self-esteem can result in negative effects on their well-being. Nevertheless, while social VR games may result in negative psychological effects in certain conditions, the net positive value of social VR can be maximized when these effects are well-understood and managed. Therefore, we assert that if game developers and managers are familiar with these potential results, social VR games can act as a constructive social medium, and the public can develop a more accurate understanding and positive view of social VR games.

## Data Availability Statement

The original contributions presented in the study are included in the article/supplementary material, further inquiries can be directed to the corresponding author.

## Ethics Statement

The studies involving human participants were reviewed and approved by the Texas A&M University Institutional Review Board. The patients/participants provided their written informed consent to participate in this study.

## Author Contributions

All authors listed have made a substantial, direct and intellectual contribution to the work, and approved it for publication.

## Funding

The open access publishing fees for this article have been furnished by the Texas A&M University Open Access to Knowledge Fund (OAKFund), supported by the University Libraries.

## Conflict of Interest

The authors declare that the research was conducted in the absence of any commercial or financial relationships that could be construed as a potential conflict of interest.

## Publisher’s Note

All claims expressed in this article are solely those of the authors and do not necessarily represent those of their affiliated organizations, or those of the publisher, the editors and the reviewers. Any product that may be evaluated in this article, or claim that may be made by its manufacturer, is not guaranteed or endorsed by the publisher.

## References

[ref1] Baumeister R. F. Campbell J. D. Krueger J. I. Vohs K. D. (2003). Does high self-esteem cause better performance, interpersonal success, happiness, or healthier lifestyles? Psychol. Sci. Public Interest 4, 1–44. doi: 10.1111/1529-1006.01431, PMID: 26151640

[ref2] Beaton A. A. Funk D. C. (2008). An evaluation of theoretical frameworks for studying physically active leisure. Leis. Sci. 30, 53–70. doi: 10.1080/01490400701756410

[ref4] Cacioppo J. T. Hawkley L. C. Thisted R. A. (2010). Perceived social isolation makes me sad: 5-year cross-lagged analyses of loneliness and depressive symptomatology in the Chicago health, aging, and social relations study. Psychol. Aging 25, 453–463. doi: 10.1037/a0017216, PMID: 20545429 PMC2922929

[ref5] Cain M. K. Zhang Z. Yuan K. H. (2017). Univariate and multivariate skewness and kurtosis for measuring nonnormality: prevalence, influence and estimation. Behav. Res. Methods. 49, 1716–35. doi: 10.3758/s13428-016-0814-1 27752968

[ref6] Cardak M. (2013). Psychological well-being and internet addiction among university students. Turkish Online J. Educ. Technol. 12, 134–141.

[ref7] Carras M. C. Van Rooij A. J. Van de Mheen D. Musci R. Xue Q. L. Mendelson T. (2017). Video gaming in a hyperconnected world: a cross-sectional study of heavy gaming, problematic gaming symptoms, and online socializing in adolescents. Comput. Hum. Behav. 68, 472–479. doi: 10.1016/j.chb.2016.11.060, PMID: 28260834 PMC5330315

[ref8] Chaplin T. M. (2006). Anger, happiness, and sadness: associations with depressive symptoms in late adolescence. J. Youth Adolesc. 35, 977–986. doi: 10.1007/s10964-006-9033-x

[ref9] Cowan K. Ketron S. (2019). A dual model of product involvement for effective virtual reality: the roles of imagination, co-creation, telepresence, and interactivity. J. Bus. Res. 100, 483–492. doi: 10.1016/j.jbusres.2018.10.063

[ref10] Diener E. Lucas R. E. Oishi S. (2002). “Subjective well-being: the science of happiness and life satisfaction,” in Handbook of Positive Psychology. *Vol*. 2. eds. Snyder C. R. Lopez S. J. (Oxford, England: Oxford University Press), 63–73.

[ref11] Diener E. Suh E. M. Lucas R. E. Smith H. L. (1999). Subjective well-being: three decades of progress. Psychol. Bull. 125, 276–302. doi: 10.1037/0033-2909.125.2.276

[ref12] Diener E. Wirtz D. Tov W. Kim-Prieto C. Choi D. W. Oishi S.. (2010). New well-being measures: short scales to assess flourishing and positive and negative feelings. Soc. Indic. Res. 97, 143–156. doi: 10.1007/s11205-009-9493-y

[ref13] Fredrickson B. L. Grewen K. M. Algoe S. B. Firestine A. M. Arevalo J. M. Ma J.. (2015). Psychological well-being and the human conserved transcriptional response to adversity. PLoS One 10:e0121839. doi: 10.1371/journal.pone.0121839, PMID: 25811656 PMC4374902

[ref14] Groves C. L. Anderson C. A. (2015). “Negative effects of video game play,” in Handbook of Digital Games and Entertainment Technologies. eds Nakatsu R. Rauterberg M. Ciancarini P. (Singapore: Springer), 1297–1322.

[ref15] Hayes A. F. (2018). Partial, conditional, and moderated moderated mediation: quantification, inference, and interpretation. Commun. Monogr. 85, 4–40. doi: 10.1080/03637751.2017.1352100

[ref16] Holt-Lunstad J. (2018). Why social relationships are important for physical health: a systems approach to understanding and modifying risk and protection. Annu. Rev. Psychol. 69, 437–458. doi: 10.1146/annurev-psych-122216-011902, PMID: 29035688

[ref17] Howard K. I. Lueger R. J. Maling M. S. Martinovich Z. (1993). A phase model of psychotherapy outcome: causal mediation of change. J. Consult. Clin. Psychol. 61, 678–685. doi: 10.1037/0022-006X.61.4.678, PMID: 8370864

[ref18] Jin S. A. (2009). Modality effects in second life: the mediating role of social presence and the moderating role of product involvement. Cyberpsychol. Behav. 12, 717–721. doi: 10.1089/cpb.2008.0273, PMID: 19522681

[ref19] Jose P. E. Ryan N. Pryor J. (2012). Does social connectedness promote a greater sense of well-being in adolescence over time? J. Res. Adolesc. 22, 235–251. doi: 10.1111/j.1532-7795.2012.00783.x

[ref20] Kernis M. H. (2003). Toward a conceptualization of optimal self-esteem. Psychol. Inq. 14, 1–26. doi: 10.1207/S15327965PLI1401_01

[ref21] King D. L. Delfabbro P. H. (2016). The cognitive psychopathology of internet gaming disorder in adolescence. J. Abnorm. Child Psychol. 44, 1635–1645. doi: 10.1007/s10802-016-0135-y, PMID: 26875565

[ref22] Lafreniere M. A. Vallerand R. J. Donahue E. G. Lavigne G. L. (2009). On the costs and benefits of gaming: the role of passion. Cyberpsychol. Behav. 12, 285–290. doi: 10.1089/cpb.2008.0234, PMID: 19366320

[ref502] LaRose R. Lin C. A. Eastin M. S. (2003). Unregulated Internet usage: addiction, habit, or deficient self-regulation? Media Psychol. 5, 225–253. doi: 10.1207/S1532785XMEP0503_01

[ref24] Lavoie R. Main K. King C. King D. (2021). Virtual experience, real consequences: the potential negative emotional consequences of virtual reality gameplay. Virtual Reality 25, 69–81. doi: 10.1007/s10055-020-00440-y

[ref25] Lee R. M. Dean B. L. Jung K. R. (2008). Social connectedness, extraversion, and subjective well-being: testing a mediation model. Personal. Individ. Differ. 45, 414–419. doi: 10.1016/j.paid.2008.05.017

[ref26] Lee R. M. Draper M. Lee S. (2001). Social connectedness, dysfunctional interpersonal behaviors, and psychological distress: testing a mediator model. J. Couns. Psychol. 48, 310–318. doi: 10.1037/0022-0167.48.3.310

[ref27] Lin C. C. (2015). Gratitude and depression in young adults: the mediating role of self-esteem and well-being. Personal. Individ. Differ. 87, 30–34. doi: 10.1016/j.paid.2015.07.017

[ref28] Liu M. Peng W. (2009). Cognitive and psychological predictors of the negative outcomes associated with playing MMOGs (massively multiplayer online games). Comput. Hum. Behav. 25, 1306–1311. doi: 10.1016/j.chb.2009.06.002

[ref29] Lo S. K. Wang C. C. Fang W. (2005). Physical interpersonal relationships and social anxiety among online game players. Cyberpsychol. Behav. 8, 15–20. doi: 10.1089/cpb.2005.8.15, PMID: 15738689

[ref30] Loton D. Borkoles E. Lubman D. Polman R. (2016). Video game addiction, engagement and symptoms of stress, depression and anxiety: the mediating role of coping. Int. J. Ment. Heal. Addict. 14, 565–578. doi: 10.1007/s11469-015-9578-6

[ref31] Mahan J. E. III Seo W. J. Jordan J. S. Funk D. (2015). Exploring the impact of social networking sites on running involvement, running behavior, and social life satisfaction. Sport Manage. Rev. 18, 182–192. doi: 10.1016/j.smr.2014.02.006

[ref33] Mandryk R. L. Frommel J. Armstrong A. Johnson D. (2020). How passion for playing world of Warcraft predicts in-game social capital, loneliness, and wellbeing. Front. Psychol. 11:2165. doi: 10.3389/fpsyg.2020.02165, PMID: 33071843 PMC7533578

[ref34] Maras D. Flament M. F. Murray M. Buchholz A. Henderson K. A. Obeid N.. (2015). Screen time is associated with depression and anxiety in Canadian youth. Prev. Med. 73, 133–138. doi: 10.1016/j.ypmed.2015.01.029, PMID: 25657166

[ref35] Martončik M. Lokša J. (2016). Do world of Warcraft (MMORPG) players experience less loneliness and social anxiety in online world (virtual environment) than in real world (offline)? Comput. Hum. Behav. 56, 127–134. doi: 10.1016/j.chb.2015.11.035

[ref36] Matthews T. Danese A. Wertz J. Odgers C. L. Ambler A. Moffitt T. E.. (2016). Social isolation, loneliness and depression in young adulthood: a behavioural genetic analysis. Soc. Psychiatry Psychiatr. Epidemiol. 51, 339–348. doi: 10.1007/s00127-016-1178-7, PMID: 26843197 PMC4819590

[ref37] McClelland D. (1988). Human Motivation. Cambridge: Cambridge University Press.

[ref38] Morgan C. Cotten S. R. (2003). The relationship between internet activities and depressive symptoms in a sample of college freshmen. Cyberpsychol. Behav. 6, 133–142. doi: 10.1089/109493103321640329, PMID: 12804025

[ref39] Orth U. Robins R. W. Roberts B. W. (2008). Low self-esteem prospectively predicts depression in adolescence and young adulthood. J. Pers. Soc. Psychol. 95, 695–708. doi: 10.1037/0022-3514.95.3.695, PMID: 18729703

[ref40] Penedo F. J. Dahn J. R. (2005). Exercise and well-being: a review of mental and physical health benefits associated with physical activity. Curr. Opin. Psychiatry 18, 189–193. doi: 10.1097/00001504-200503000-00013, PMID: 16639173

[ref41] Pressman S. D. Jenkins B. N. Moskowitz J. T. (2019). Positive affect and health: what do we know and where next should we go? Annu. Rev. Psychol. 70, 627–650. doi: 10.1146/annurev-psych-010418-102955, PMID: 30260746

[ref42] Radloff L. S. (1991). The use of the Center for Epidemiologic Studies Depression Scale in adolescents and young adults. J. Youth Adolesc. 20, 149–166. doi: 10.1007/BF01537606, PMID: 24265004

[ref43] Rosenberg M. (2015). Society and the Adolescent Self-Image. New Jersey: Princeton University Press.

[ref44] Schmidt J. A. Padilla B. (2003). Self-esteem and family challenge: an investigation of their effects on achievement. J. Youth Adolesc. 32, 37–46. doi: 10.1023/A:1021080323230

[ref45] Sedikides C. Gregg A. P. Cisek S. Hart C. M. (2007). The I that buys: narcissists as consumers. J. Consum. Psychol. 17, 254–257. doi: 10.1016/S1057-7408(07)70035-9

[ref46] Singh D. K. Rahman N. N. Seffiyah R. Chang S. Y. Zainura A. K. Aida S. R.. (2017). Impact of virtual reality games on psychological well-being and upper limb performance in adults with physical disabilities: a pilot study. Med. J. Malaysia 72, 119–121. PMID: 28473675

[ref47] Smyth J. M. (2007). Beyond self-selection in video game play: an experimental examination of the consequences of massively multiplayer online role-playing game play. Cyberpsychol. Behav. 10, 717–721. doi: 10.1089/cpb.2007.9963, PMID: 17927543

[ref48] Stetina B. U. Kothgassner O. D. Lehenbauer M. Kryspin-Exner I. (2011). Beyond the fascination of online-games: probing addictive behavior and depression in the world of online-gaming. Comput. Hum. Behav. 27, 473–479. doi: 10.1016/j.chb.2010.09.015

[ref49] Tavakol M. Dennick R. (2011). Making sense of Cronbach’s alpha. Int. J. Med. Educ. 2, 53–55. doi: 10.5116/ijme.4dfb.8dfd, PMID: 28029643 PMC4205511

[ref50] Uhm J.-P. Lee H.-W. Han J.-W. (2020). Creating sense of presence in a virtual reality experience: impact on neurophysiological arousal and attitude towards a winter sport. Sport Manage. Rev. 23, 588–600. doi: 10.1016/j.smr.2019.10.003

[ref51] Valkenburg P. M. Peter J. Walther J. B. (2016). Media effects: theory and research. Annu. Rev. Psychol. 67, 315–338. doi: 10.1146/annurev-psych-122414-033608, PMID: 26331344

[ref53] Wang J. L. Sheng J. R. Wang H. Z. (2019). The association between mobile game addiction and depression, social anxiety, and loneliness. Front. Public Health 7:247. doi: 10.3389/fpubh.2019.00247, PMID: 31552213 PMC6743417

[ref54] Williams K. L. Galliher R. V. (2006). Predicting depression and self–esteem from social connectedness, support, and competence. J. Soc. Clin. Psychol. 25, 855–874. doi: 10.1521/jscp.2006.25.8.855

[ref55] Williams A. L. Merten M. J. (2008). A review of online social networking profiles by adolescents: implications for future research and intervention. Adolescence 43, 253–274. PMID: 18689100

[ref56] World Health Organization (2018). Gaming disorder. Available at: http://www.who.int/features/qa/gaming-disorder/en/ (Accessed August 1, 2021).

[ref57] Zaichkowsky J. L. (1985). Measuring the involvement construct. J. Consum. Res. 12, 341–352. doi: 10.1086/208520

[ref501] Zaichkowsky J. L. (1994). The personal involvement inventory: reduction, revision, and application to advertising. J. Advert. 23, 59–70. doi: 10.1080/00913367.1943.10673459

